# Supplementary material for: Caries Prevalence Associated with Oral Health-Related Behaviors among Romanian Schoolchildren
Source: Int J Environ Res Public Health. 2021 Jun 17;18(12):6515. doi: 10.3390/ijerph18126515 (PMC8296518; doi:10.3390/ijerph18126515)
Supplement: Supplementary file 1 [file ijerph-18-06515-s001.zip › ijerph-1266874-supplementary.pdf]

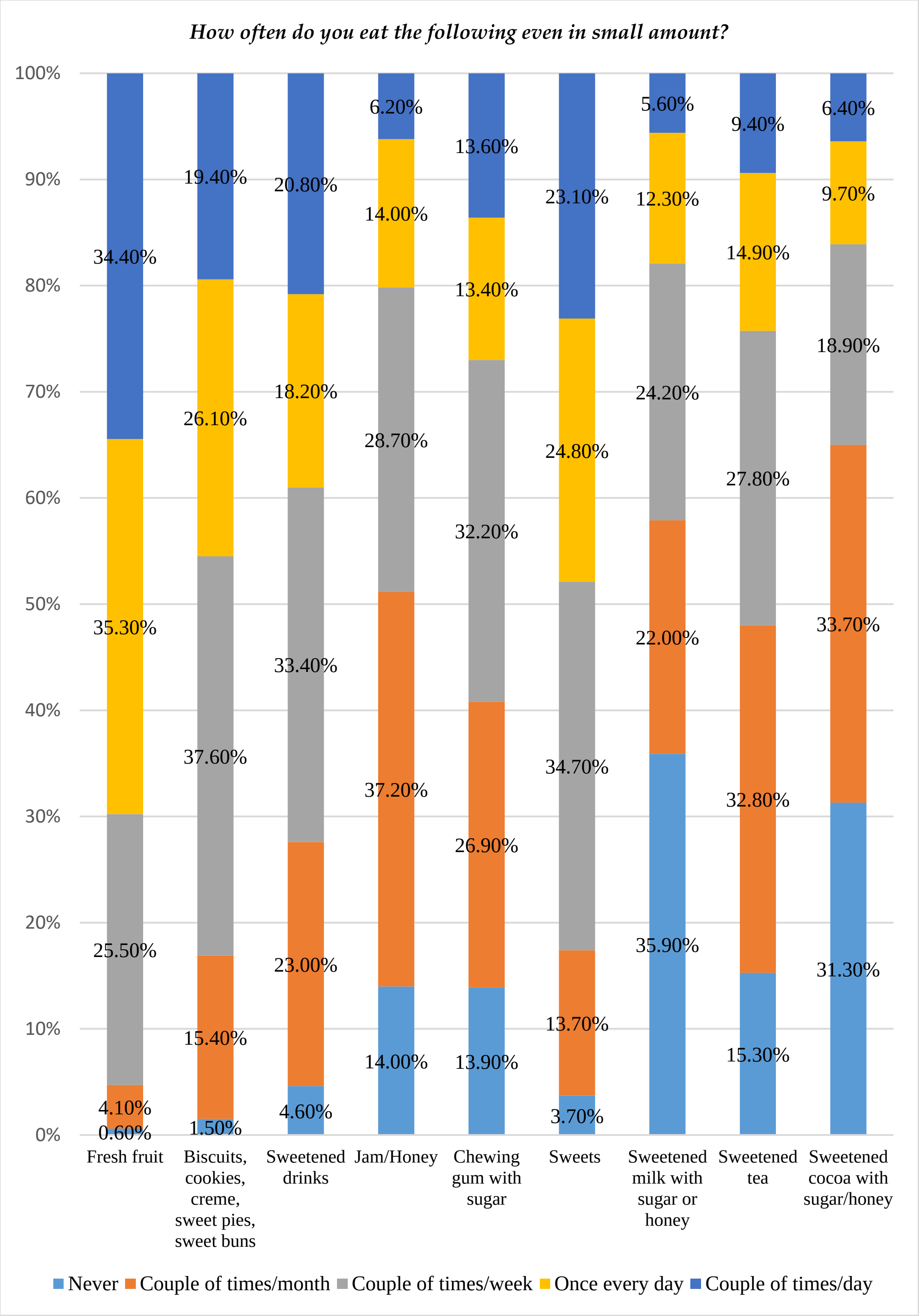

Figure S1. *Eating behavior of 12 years old children.*

Table S1. *Correlation between fresh fruit consumption and children's demographics*

Fresh fruit	Gender	Father's education	Mother's education	Residence	Type of city
Pearson Correlation	0.00	- 0.03	0.03	- 0.06	- 0.00
Sig0. (2-tailed)	0.95	0.29	0.31	0.07	0.9
N	804	802	801	807	807

Table S2. *Correlation between the consumption of biscuits, cakes, cream, sweet pies, buns and the demographic data of children*

**Biscuits, cookies, creme, sweet pies, sweet buns**	**Gender**	**Father's education**	**Mother's education**	**Residence**	**Type of city**
Pearson Correlation	0.03	- 0.08*	- 0.10**	- 0.09**	0.00
Sig0. (2-tailed)	0.27	0.02	0.00	0.00	0.88
N	797	795	794	800	800

Table S3. *Correlation between the consumption of sweetened soft drinks and the demographic data of children.*

**Sweetened drinks**	**Gender**	**Father's education**	**Mother's education**	**Residence**	**Type of city**
Pearson Correlation	- 0.10**	- 0.14**	- 0.18**	- 0.18**	- 0.17**
Sig0. (2-tailed)	0.00	0.00	0.00	0.00	0.00
N	800	798	797	803	803

Table S4. *Correlation between jam / honey consumption and demographic data of children.*

**Jam/Honey**	**Gender**	**Father's education**	**Mother's education**	**Residence**	**Type of city**
Pearson Correlation	- 0.03	- 0.06	- 0.01	0.11**	- 0.09*
Sig0. (2-tailed)	0.32	0.06	0.68	0.00	0.01
N	799	797	796	802	802

Table S5. *Correlation between sugar consumption of chewing gum and children's demographics.*

**Chewing gum with sugar**	**Gender**	**Father's education**	**Mother's education**	**Residence**	**Type of city**
Pearson Correlation	0.04	- 0.06	- 0.16**	- 0.08**	- 0.06
Sig0. (2-tailed)	0.22	0.08	0.00	0.01	0.06
N	801	799	798	804	804

Table S6. *Correlation between the consumption of sweets / candies and the demographic data of children.*

**Sweets/ candies**	**Gender**	**Father's education**	**Mother's education**	**Residence**	**Type of city**
Pearson Correlation	0.04	- 0.12**	- 0.16**	- 0.13**	- 0.09**
Sig0. (2-tailed)	0.16	0.00	0.00	0.00	0.00
N	802	800	799	805	805

Table S7. *Correlation between milk / sugar / honey consumption and children's demographics.*

Sweetented milk with sugar/honey	Gender	Father's education	Mother's education	Residence	Type of city
Pearson Correlation	- 0.04	- 0.00	0.04	- 0.10**	- 0.05
Sig0. (2-tailed)	0.19	0.90	0.22	0.00	0.11
N	796	79	793	799	799

Table S8. *Correlation between sweetened tea consumption and demographic data of children.*

Sweetened tea	Gender	Father's education	Mother's education	Residence	Type of city
Pearson Correlation	0.01	- 0.05	- 0.09**	- 0.06	- 0.09**
Sig0. (2-tailed)	0.71	0.16	0.00	0.06	0.00
N	797	795	794	800	800

Table S9. *Correlation between cocoa sugar / honey consumption and children's demographics.*

Sweetened cocoa with sugar/honey	Gender	Father's education	Mother's education	Residence	Type of city
Pearson Correlation	- 0.05	- 0.04	- 0.09**	- 0.05	- 0.02
Sig0. (2-tailed)	0.12	0.26	0.00	0.14	0.40
N	798	796	795	801	801
